# Supplementary material for: Raman image-activated cell sorting
Source: Nat Commun. 2020 Jul 10;11:3452. doi: 10.1038/s41467-020-17285-3 (PMC7351993; doi:10.1038/s41467-020-17285-3)
Supplement: Supplementary file 1 — Supplementary Information [file 41467_2020_17285_MOESM1_ESM.docx]

**Supplementary Information**

for

**Raman image-activated cell sorting**

by Nitta et al.

This file contains the following materials:

- Supplementary Figure 1
- Supplementary Figure 2
- Supplementary Figure 3
- Supplementary Figure 4
- Supplementary Figure 5
- Supplementary Figure 6
- Supplementary Figure 7
- Supplementary Figure 8
- Supplementary Figure 9
- Supplementary Table 1

*
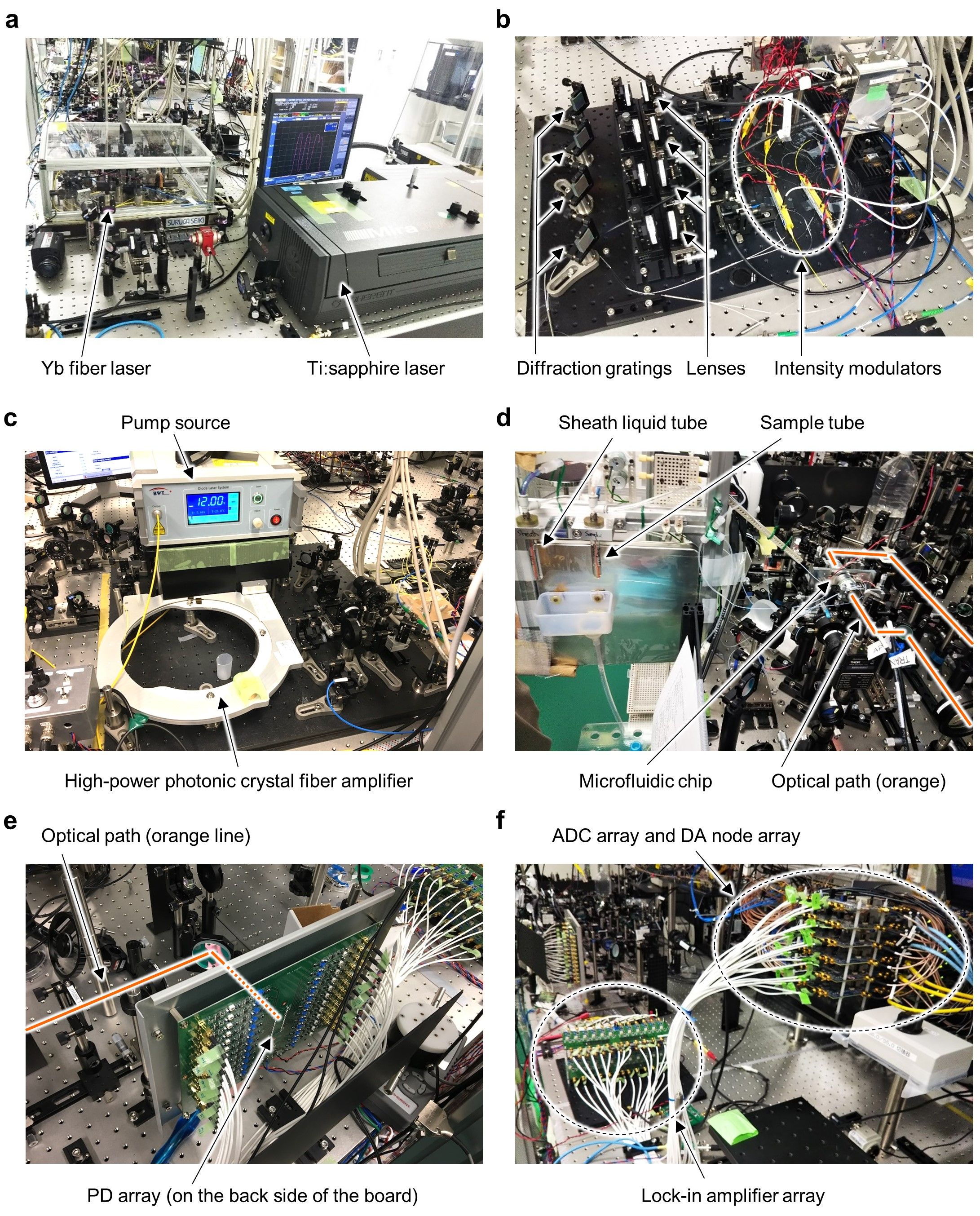
*

**Supplementary Figure 1 | Pictures of the major components of the RIACS. a,** Ti:sapphire laser and Yb fiber laser. **b,** Four-color band-pass filter for generating pulse-pair-resolved wavelength-switched pulses. **c,** High-power gain module composed of a pump source and a high-power photonic crystal fiber amplifier. **d,** Sample tube, sheath liquid tube, and optics around the microfluidic chip. **e,** 24-ch PD array circuit. **f,** Lock-in amplifier array, ADC array, and DA node array.


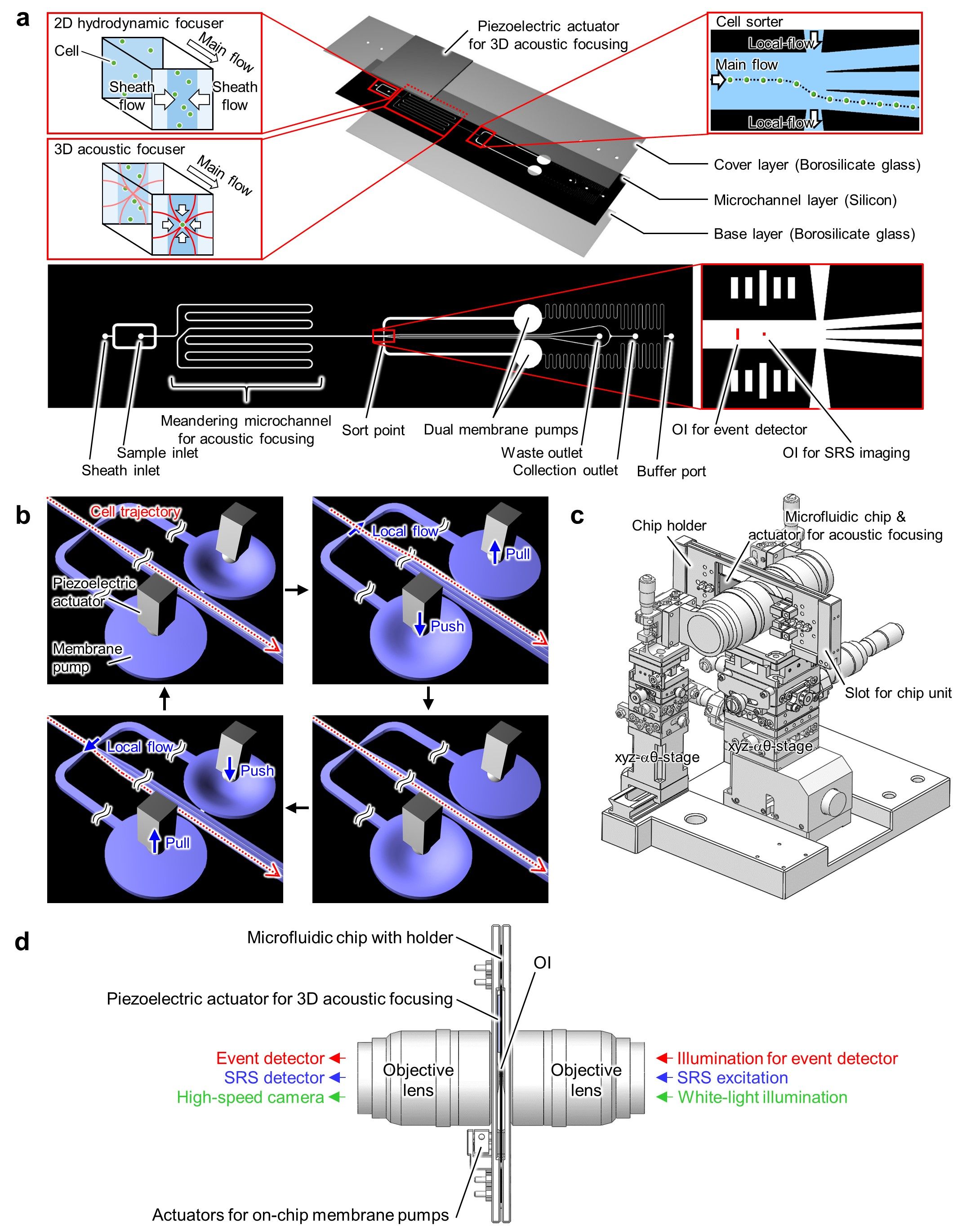


**Supplementary Figure 2 | Details of the microfluidic chip, on-chip dual-membrane push-pull cell sorter, and optics-microfluidics integration unit. a,** Schematic of the microfluidic chip that contains the hydrodynamic focuser, acoustic focuser, optical interrogation (OI) area (including an OI for the event detector and an OI for the SRS microscope), and dual-membrane push-pull cell sorter. **b,** Working principles of the on-chip dual-membrane push-pull cell sorter. **c,** Schematic of the optics-microfluidics integration unit. **d,** Interface of the optics-microfluidics integration unit with the OI points and optical beams.


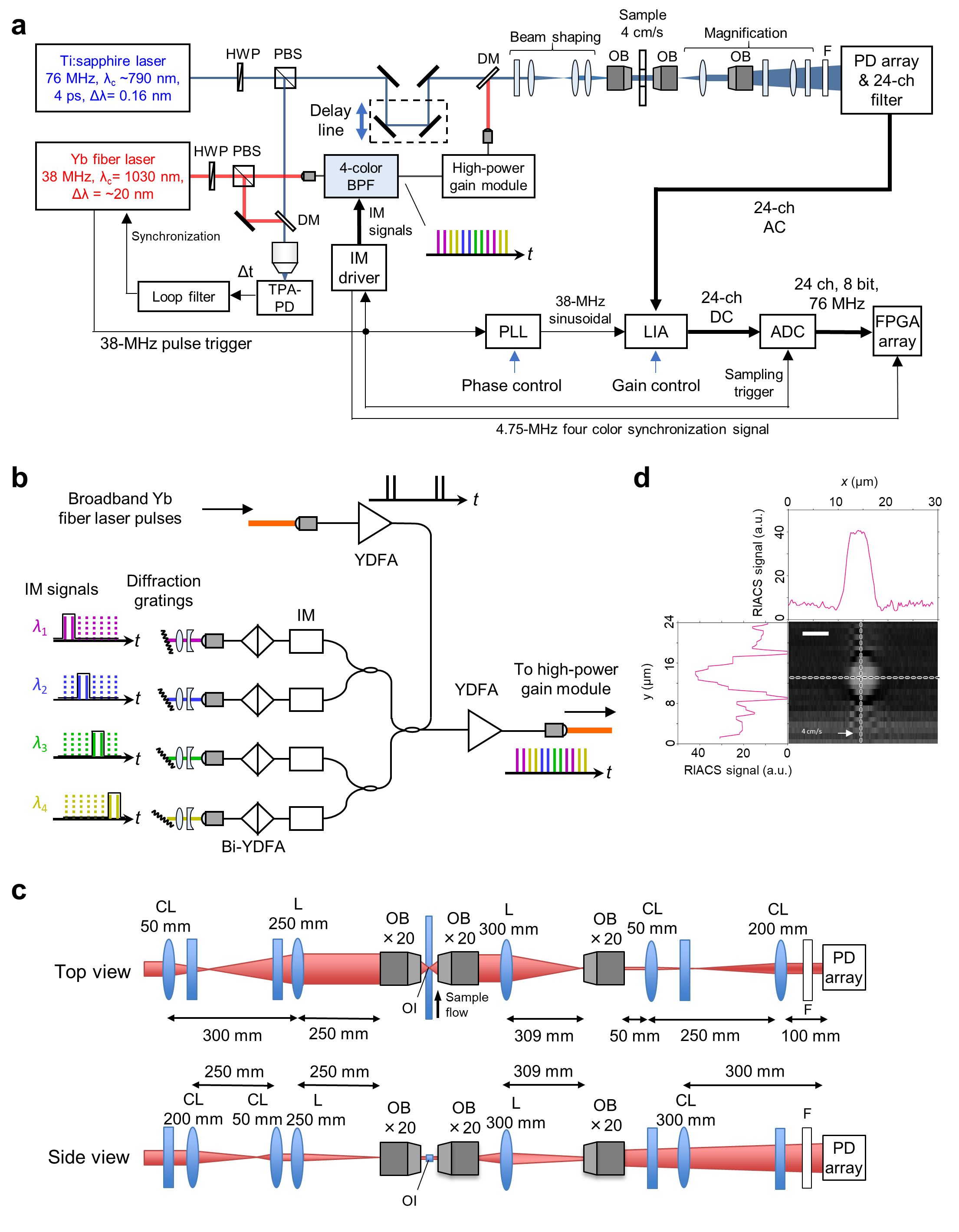


**Supplementary Figure 3 | Details of the ultrafast multicolor SRS microscope. a,** Schematic of the microscope. HWP, half wave plate; PBS, polarizing beam splitter; TPA-PD, two-photon absorption photodiode; DM, dichroic mirror; OB, objective lens; F, short-pass filter; PLL, phase-locked loop; LIA, lock-in amplifier. **b,** Schematic of the pulse-pair-resolved wavelength-switchable laser. **c,** Schematic of the beam shaper. **d,** Cross sections of the RIACS image of a PS particle in the directions parallel (*x*) and perpendicular (*y*) to the flow (representative of *n* = 3). Scale bar: 5 µm.


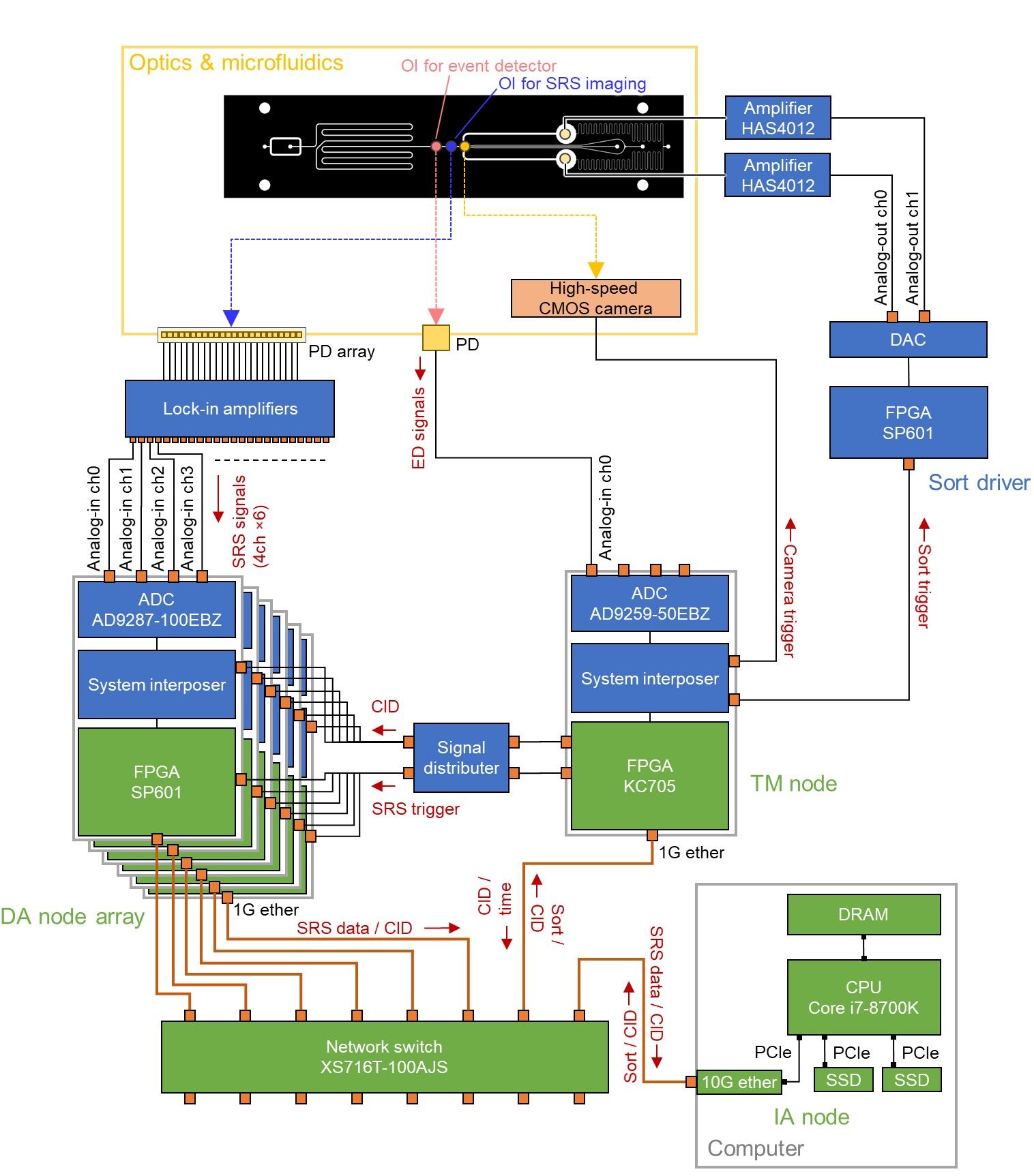


**Supplementary Figure 4 | Details of the real-time Raman image processor.** The real-time Raman image processor is based on a hybrid FPGA-CPU infrastructure on a 10-Gbps all-IP network that enables high scalability, high throughout, high flexibility, and real-time automated operation for digital image processing and decision making. DAC, digital-to-analog converter; DRAM, dynamic random-access memory.


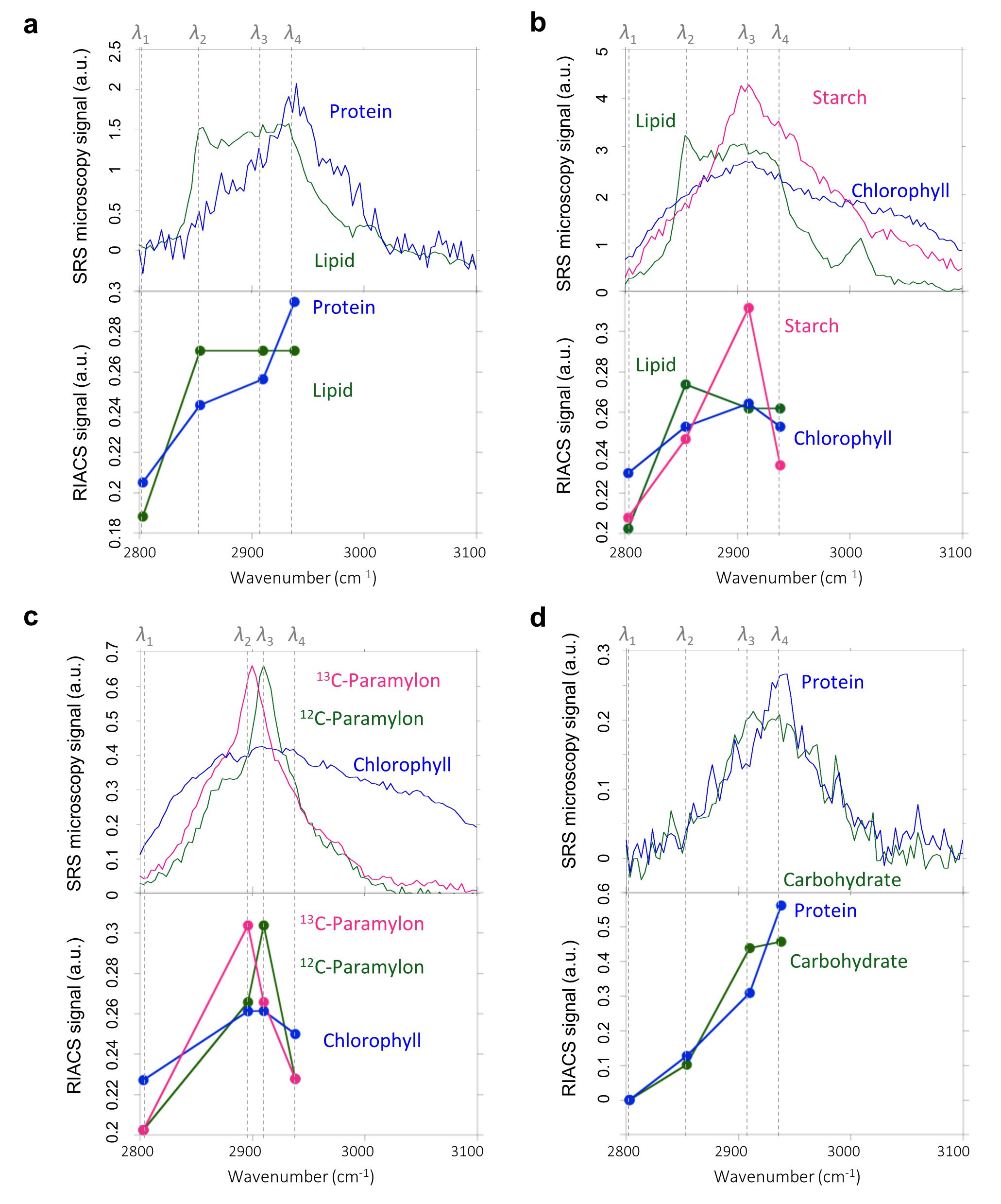


**Supplementary Figure 5 | Selection of the wavenumbers.** Four-color SRS spectra obtained by the SRS microscope and SRS spectra taken independently from (**a**) 3T3-L1 cells, (**b**) *Chlamydomonas* sp. KC4 cells, (**c**) ^12^C- or ^13^C-probed *Euglena gracilis* cells, and (**d**) hiPSCs cells. In these figure panels, each SRS spectrum was adjusted to the four-color SRS spectrum for comparison. The spectrum attributed to protein in panel (**a**) was enlarged by a factor of 3.7.


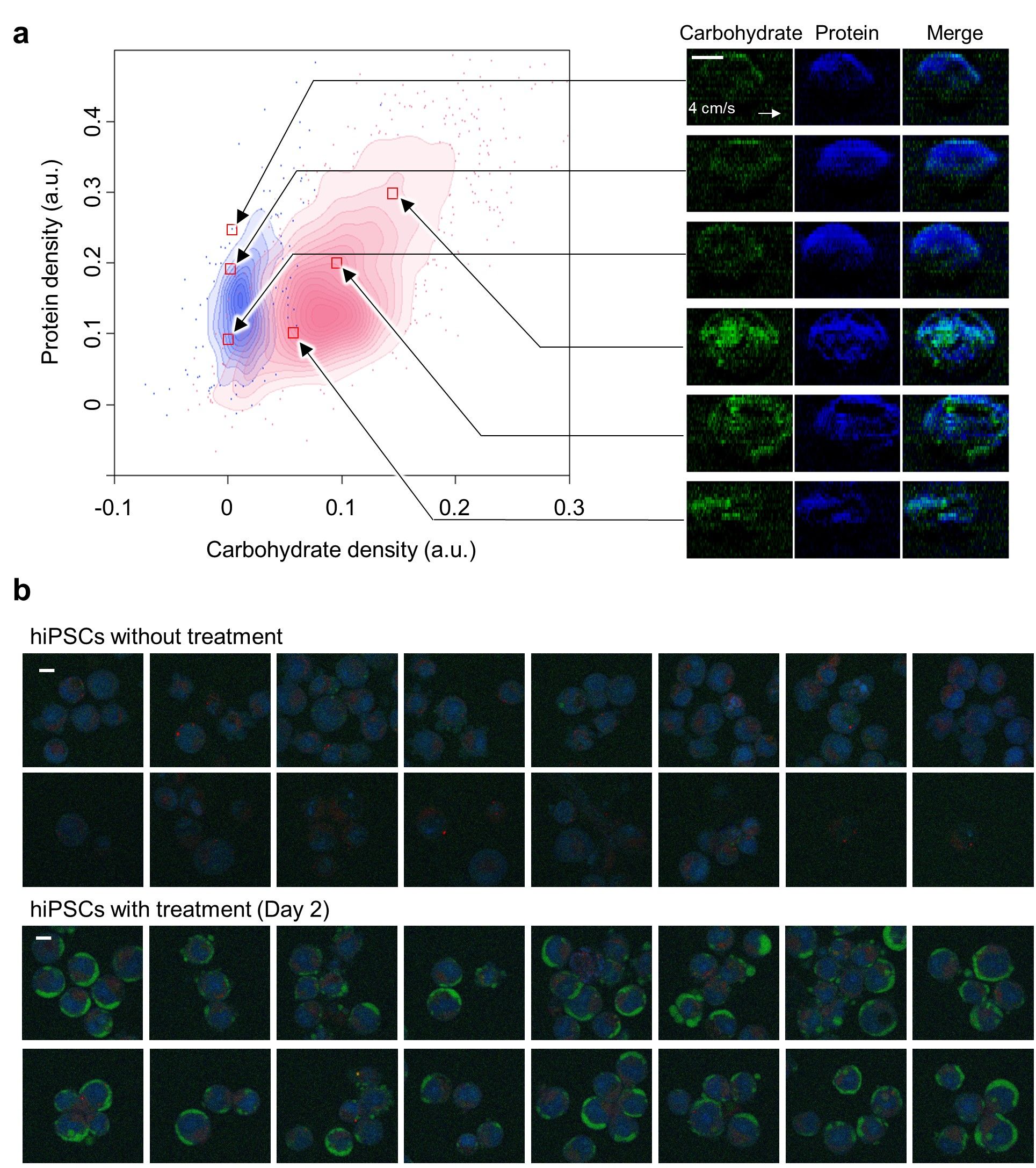


**Supplementary Figure 6 | Comparison between hiPSCs in two different culture media for naïve and primed pluripotent states. a,** Scatter plot of hiPSCs (red for the naïve pluripotent state, *n* = 3,608, and blue for the primed pluripotent state, *n* = 620). The inset shows SRS images of hiPSCs at the specified locations in the scatter plot. Scale bar: 10 µm. **b,** SRS microscope images of these cultures (red, blue, and green for lipids, protein, and carbohydrates, respectively, *n* = 16 for each culture). Scale bar: 10 µm.

**
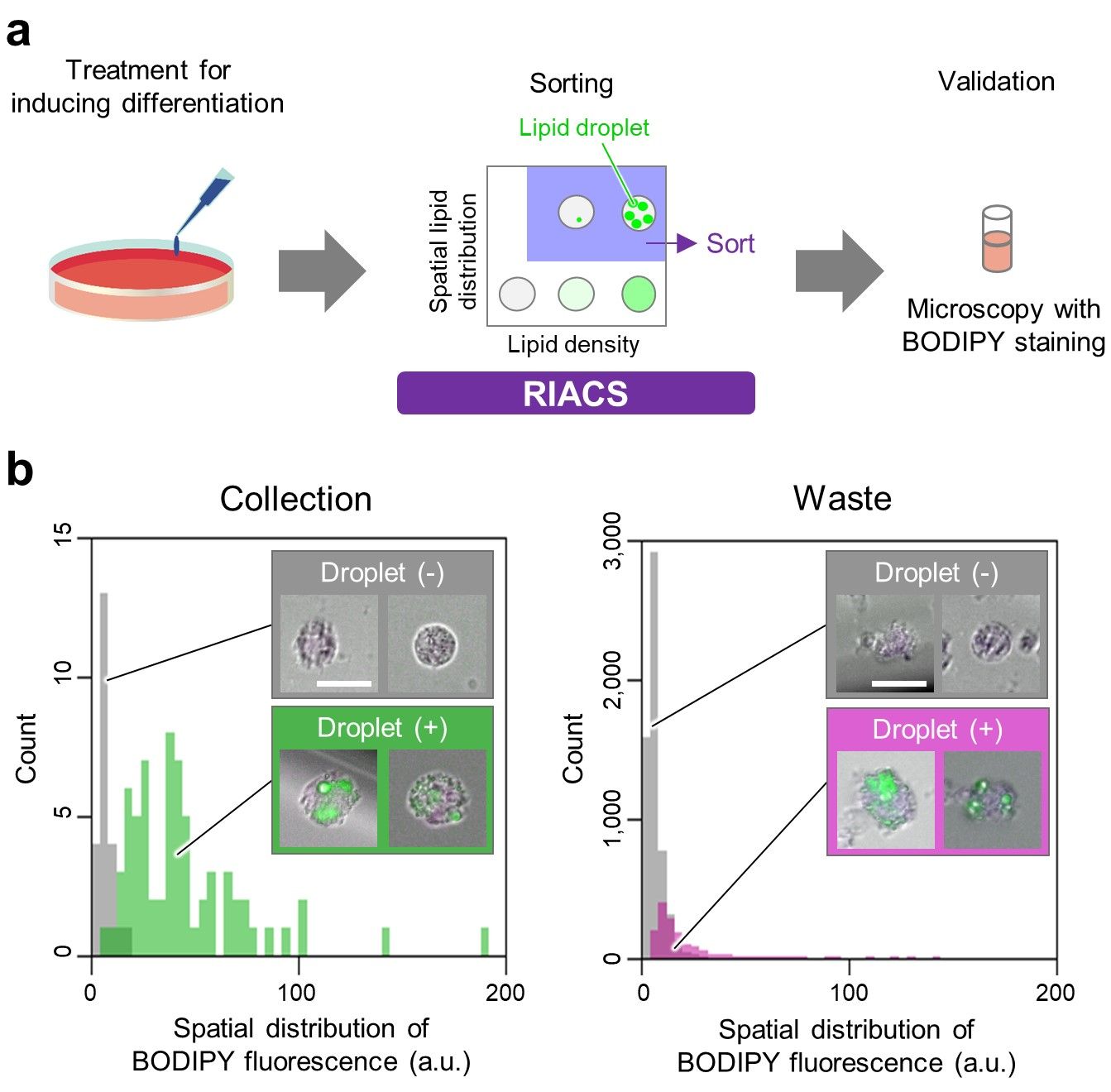
**

**Supplementary Figure 7 | Raman image-activated sorting of adipocyte-like cells.** **a,** Experimental procedure. 3T3-L1 cells were induced to differentiate into adipocyte-like cells, sorted by the RIACS in a label-free manner to enrich cells with a large spatial distribution of cytoplasmic lipid droplets, and validated under a fluorescence microscope. **b,** Histograms of sorted and unsorted 3T3-L1 cells in the collection and waste tubes, respectively, in the spatial distribution of BODIPY fluorescence spots. The insets show fluorescence images of typical sorted and unsorted cells. All the cell images were manually classified into two groups: cells with and without lipid droplets (droplet+ and droplet-, respectively). For the sorted cells, *n* = 67 and 23 are for droplet+ and droplet-, respectively. For the unsorted cells, *n* = 1,400 and 5,755 are for droplet+ and droplet-, respectively. Scale bar: 20 µm.


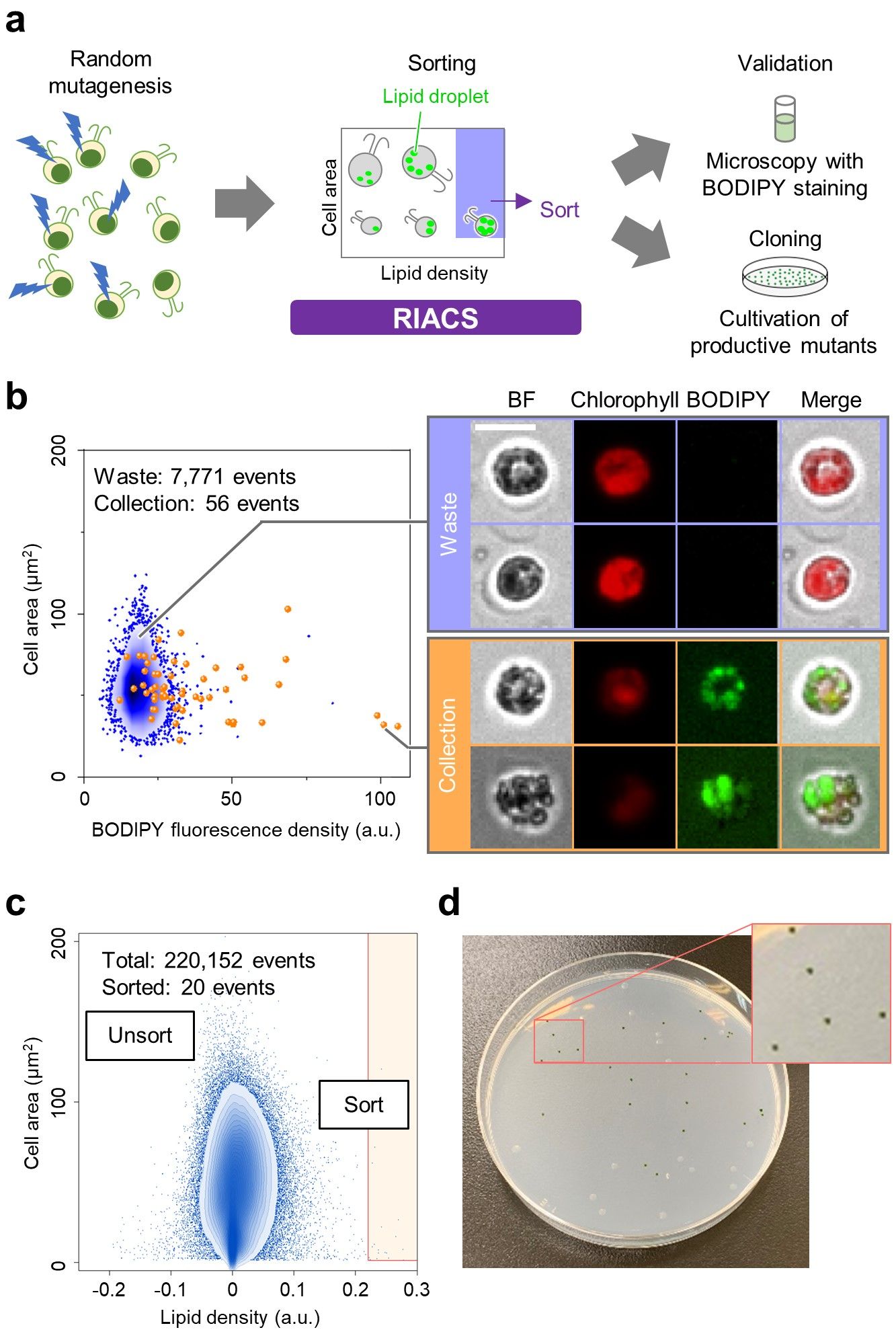


**Supplementary Figure 8 | Raman image-activated sorting of *Chlamydomonas* sp. cells.** **a,** Experimental procedure. *Chlamydomonas* sp. KC4 cells were transformed by plasma irradiation for random mutagenesis, sorted by the RIACS in a label-free manner to isolate highly lipid-rich cells, and cloned or validated under a fluorescence microscope. **b,** Scatter plot of sorted (orange) and unsorted (blue) cells in the collection and waste tubes, respectively, in cell area and fluorescence density. The inset shows representative fluorescence images of sorted and unsorted cells (*n* = 56 and 7,715, respectively). BF; Bright field. Scale bar: 10 µm. **c,** Scatter plot of *Chlamydomonas* sp. KC4 cells in cell area and lipid density, with the sort region (yellow). Extremely rare, super-lipid-rich mutants that constitute about 0.009% of the total population were sorted by the RIACS. **d,** Colonies of the sorted mutants in panel **c**.


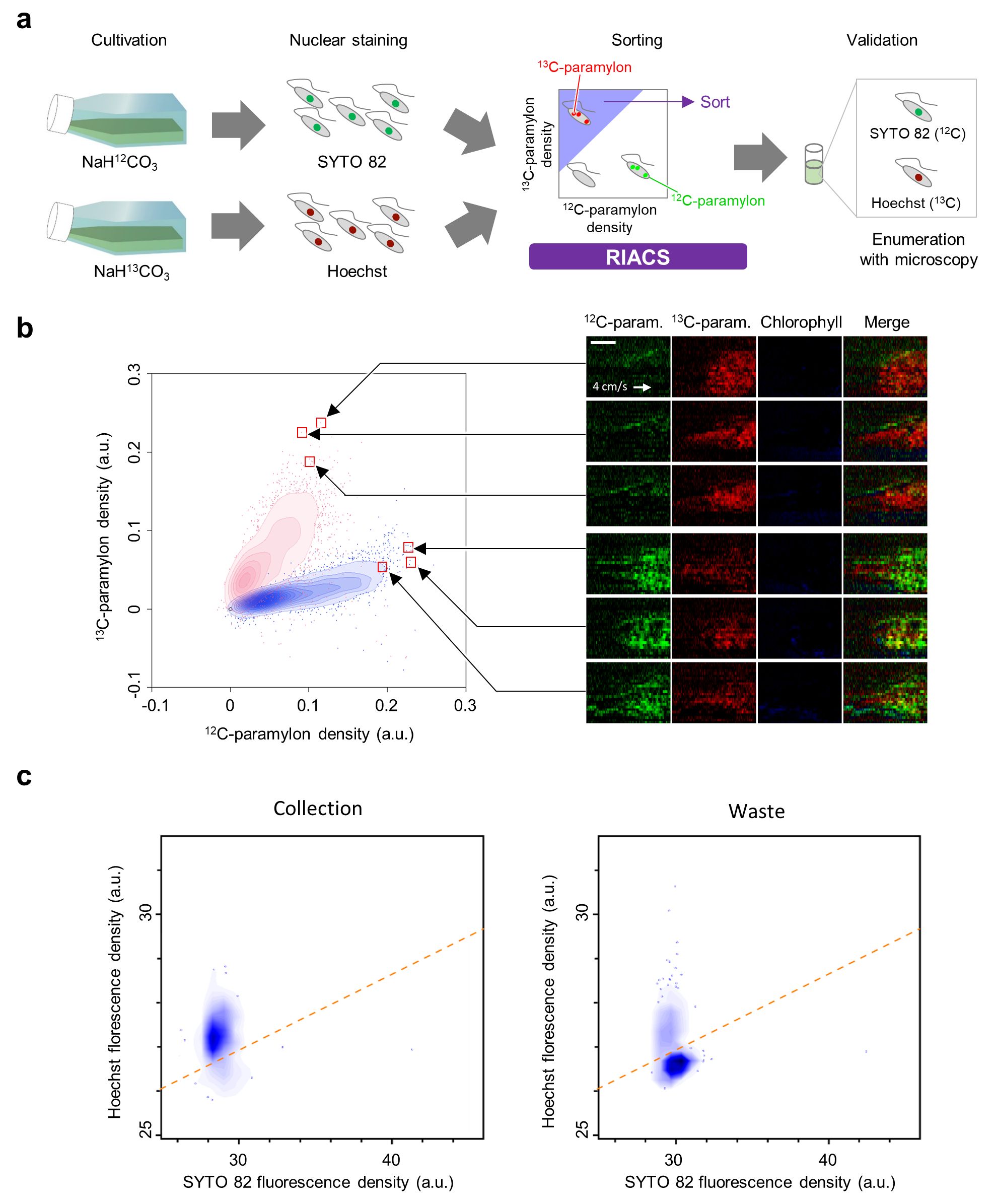


**Supplementary Figure 9 | Raman image-activated sorting of *Euglena gracilis* cells. a,** Experimental procedure. *Euglena gracilis* cells were cultivated in culture media containing NaH^12^CO_3_ or NaH^13^CO_3_ for SIP, stained with fluorescent labels, sorted by the RIACS, and validated under a fluorescence microscope. **b,** Scatter plots of independently measured *Euglena gracilis* cells cultivated in the two different culture media, in ^12^C- and ^13^C-paramylon intensity per cell area. The insets show representative SRS images of cells cultured in the NaH^12^CO_3_ media (*n* = 5,216) and NaH^13^CO_3_ media (*n* = 5,273). Scale bar: 10 µm. **c,** Scatter plots of sorted and unsorted cells in the collection and waste tubes, respectively, in fluorescence intensity.

**Supplementary Table 1 | Sample space and expected sort results.** The size of the sample space is 32. Particle A, particle following the particle of interest; Particle B, particle preceding the particle of interest; Interval A, interval between Particle A and the particle of interest; Interval B; interval between Particle B and the particle of interest; TP, true positive; FP, false positive; TN, true negative; FN, false negative; *P*_p_, *P*_p_ (*T* < *τ*).

| Scenario | Particle A | Interval A | Particle of interest | Interval B | Particle B | Sort result |
| --- | --- | --- | --- | --- | --- | --- |
| 1 | *r* | 1-*P*_p_ | *r* | 1-*P*_p_ | *r* | TP |
| 2 | *r* | 1-*P*_p_ | *r* | 1-*P*_p_ | 1-*r* | TP |
| 3 | *r* | 1-*P*_p_ | *r* | *P*_p_ | *r* | FN |
| 4 | *r* | 1-*P*_p_ | *r* | *P*_p_ | 1-*r* | TP |
| 5 | *r* | 1-*P*_p_ | 1-*r* | 1-*P*_p_ | *r* | TN |
| 6 | *r* | 1-*P*_p_ | 1-*r* | 1-*P*_p_ | 1-*r* | TN |
| 7 | *r* | 1-*P*_p_ | 1-*r* | *P*_p_ | *r* | FP |
| 8 | *r* | 1-*P*_p_ | 1-*r* | *P*_p_ | 1-*r* | TN |
| 9 | *r* | *P*_p_ | *r* | 1-*P*_p_ | *r* | FN |
| 10 | *r* | *P*_p_ | *r* | 1-*P*_p_ | 1-*r* | FN |
| 11 | *r* | *P*_p_ | *r* | *P*_p_ | *r* | TP |
| 12 | *r* | *P*_p_ | *r* | *P*_p_ | 1-*r* | FN |
| 13 | *r* | *P*_p_ | 1-*r* | 1-*P*_p_ | *r* | FP |
| 14 | *r* | *P*_p_ | 1-*r* | 1-*P*_p_ | 1-*r* | FP |
| 15 | *r* | *P*_p_ | 1-*r* | *P*_p_ | *r* | TN |
| 16 | *r* | *P*_p_ | 1-*r* | *P*_p_ | 1-*r* | FP |
| 17 | 1-*r* | 1-*P*_p_ | *r* | 1-*P*_p_ | *r* | TP |
| 18 | 1-*r* | 1-*P*_p_ | *r* | 1-*P*_p_ | 1-*r* | TP |
| 19 | 1-*r* | 1-*P*_p_ | *r* | *P*_p_ | *r* | FN |
| 20 | 1-*r* | 1-*P*_p_ | *r* | *P*_p_ | 1-*r* | TP |
| 21 | 1-*r* | 1-*P*_p_ | 1-*r* | 1-*P*_p_ | *r* | TN |
| 22 | 1-*r* | 1-*P*_p_ | 1-*r* | 1-*P*_p_ | 1-*r* | TN |
| 23 | 1-*r* | 1-*P*_p_ | 1-*r* | *P*_p_ | *r* | FP |
| 24 | 1-*r* | 1-*P*_p_ | 1-*r* | *P*_p_ | 1-*r* | TN |
| 25 | 1-*r* | *P*_p_ | *r* | 1-*P*_p_ | *r* | TP |
| 26 | 1-*r* | *P*_p_ | *r* | 1-*P*_p_ | 1-*r* | TP |
| 27 | 1-*r* | *P*_p_ | *r* | *P*_p_ | *r* | FN |
| 28 | 1-*r* | *P*_p_ | *r* | *P*_p_ | 1-*r* | TP |
| 29 | 1-*r* | *P*_p_ | 1-*r* | 1-*P*_p_ | *r* | TN |
| 30 | 1-*r* | *P*_p_ | 1-*r* | 1-*P*_p_ | 1-*r* | TN |
| 31 | 1-*r* | *P*_p_ | 1-*r* | *P*_p_ | *r* | FP |
| 32 | 1-*r* | *P*_p_ | 1-*r* | *P*_p_ | 1-*r* | TN |
