# Supplementary material for: Raman image-activated cell sorting
Source: Nat Commun. 2020 Jul 10;11:3452. doi: 10.1038/s41467-020-17285-3 (PMC7351993; doi:10.1038/s41467-020-17285-3)
Supplement: Supplementary file 3 — Description of Additional Supplementary Files [file 41467_2020_17285_MOESM3_ESM.pdf]

**Title:** Supplementary Movie 1

**Description: Raman image-activated sorting of polymer particles.** The movie of flowing polymer particles with a throughput of about 50 eps was obtained by the high-speed CMOS camera (V1211, Vision Research Inc., NJ, USA).
