# Supplementary material for: Raman image-activated cell sorting
Source: Nat Commun. 2020 Jul 10;11:3452. doi: 10.1038/s41467-020-17285-3 (PMC7351993; doi:10.1038/s41467-020-17285-3)
Supplement: Supplementary file 4 — Reporting Summary [file 41467_2020_17285_MOESM4_ESM.pdf]

nature research

Corresponding author(s): Keisuke Goda

Last updated by author(s): Jun 15, 2020

Reporting Summary

Nature Research wishes to improve the reproducibility of the work that we publish. This form provides structure for consistency and transparency in reporting. For further information on Nature Research policies, see Authors & Referees and the Editorial Policy Checklist .

Statistics

For all statistical analyses, confirm that the following items are present in the figure legend, table legend, main text, or Methods section.

n/a	Confirmed	
☐	☒	The exact sample size (*n*) for each experimental group/condition, given as a discrete number and unit of measurement
☒	☐	A statement on whether measurements were taken from distinct samples or whether the same sample was measured repeatedly
☒	☐	The statistical test(s) used AND whether they are one- or two-sided<br>*Only common tests should be described solely by name; describe more complex techniques in the Methods section.*
☒	☐	A description of all covariates tested
☒	☐	A description of any assumptions or corrections, such as tests of normality and adjustment for multiple comparisons
☒	☐	A full description of the statistical parameters including central tendency (e.g. means) or other basic estimates (e.g. regression coefficient) AND variation (e.g. standard deviation) or associated estimates of uncertainty (e.g. confidence intervals)
☒	☐	For null hypothesis testing, the test statistic (e.g. *F*, *t*, *r*) with confidence intervals, effect sizes, degrees of freedom and *P* value noted<br>*Give P values as exact values whenever suitable.*
☒	☐	For Bayesian analysis, information on the choice of priors and Markov chain Monte Carlo settings
☒	☐	For hierarchical and complex designs, identification of the appropriate level for tests and full reporting of outcomes
☒	☐	Estimates of effect sizes (e.g. Cohen's *d*, Pearson's *r*), indicating how they were calculated

Our web collection on statistics for biologists contains articles on many of the points above.

Software and code

Policy information about availability of computer code

Data collection	Ubuntu Linux 16.04, OpenCV 3. The custom codes for image analysis and sort/unsort decision making are available from a public repository (https://doi.org/10.5281/zenodo.3892192).
Data analysis	R 4.0.0, Python 3.7, ImageJ 1.52. The custom codes for the figures are available from a public repository (https://doi.org/10.5281/zenodo.3892192).

For manuscripts utilizing custom algorithms or software that are central to the research but not yet described in published literature, software must be made available to editors/reviewers. We strongly encourage code deposition in a community repository (e.g. GitHub). See the Nature Research guidelines for submitting code & software for further information.

Data

Policy information about availability of data

All manuscripts must include a data availability statement. This statement should provide the following information, where applicable:

- Accession codes, unique identifiers, or web links for publicly available datasets
- A list of figures that have associated raw data
- A description of any restrictions on data availability

The source data underlying Figures 2c, 2d, 4b, 2d, and 2f is available in the custom codes. An additional dataset that supports findings in this study is available upon reasonable request to the corresponding author (https://doi.org/10.5281/zenodo.3892192).

Field-specific reporting

Please select the one below that is the best fit for your research. If you are not sure, read the appropriate sections before making your selection.

Life sciences study design

All studies must disclose on these points even when the disclosure is negative.

Sample size	The total number of events in sorting experiments was determined to obtain at least 10 sorted cells, based on the estimated ratio of the sort target to the total population. In measurement experiments, the total number of events was set to be at least 5000. However, if it was difficult to achieve, we allowed for as few as 2000 events.
Data exclusions	Unsuccessful events for producing binary image masks due to poor image quality were excluded from analysis.
Replication	We repeated sorting experiments with multiple samples or conditions to evaluate the reproducibility of the signal processing time, sorting performance, etc. The experimental findings were reliably reproduced by at least two independent experiments.
Randomization	N/A (We did not conduct any cross-sample/organism/participant comparison work in this paper)
Blinding	N/A (We did not perform any controlled trials in this paper)

Reporting for specific materials, systems and methods

We require information from authors about some types of materials, experimental systems and methods used in many studies. Here, indicate whether each material, system or method listed is relevant to your study. If you are not sure if a list item applies to your research, read the appropriate section before selecting a response.

Materials & experimental systems

n/a	Involved in the study
☒	☐ Antibodies
☐	☒ Eukaryotic cell lines
☒	☐ Palaeontology
☒	☐ Animals and other organisms
☒	☐ Human research participants
☒	☐ Clinical data

Methods

n/a	Involved in the study
☒	☐ ChIP-seq
☒	☐ Flow cytometry
☒	☐ MRI-based neuroimaging

Eukaryotic cell lines

Policy information about cell lines

Cell line source(s)	3T3-L1 (CL-173) was obtained from ATCC. IM-E1-5 (Song L et al., 2015) was provided by M. Yazawa. Chlorella sorokiniana (TKAC1027) and Chlamydomonas reinhardtii (TKAC1017) were obtained from Algae Collection at Tsuruoka, Keio. Hamakko caudatus (NIES-22939, Gloeomonas anomalipyrenoides (NIES-3640), and Euglena gracilis (NIES-48) were obtained from Microbial Culture Collection at NIES. Chlamydomonas sp. (KC4) was provided by T. Hasunuma.
Authentication	For IM-E1-5, G-band karyotyping and in vitro differentiation potential were tested and verified to maintain this line. All other cell lines used were routinely checked for their morphology during cultivation under a light microscope. However, none of them were authenticated by genotyping or other means.
Mycoplasma contamination	IM-E1-5 was tested negative of Mycoplasma contamination. All other cell lines used were not tested for mycoplasma contamination.
Commonly misidentified lines (See ICLAC register)	No commonly misidentified cell lines were used.
